# Game statistics that discriminate winning and losing at the NBA level of basketball competition

**DOI:** 10.1371/journal.pone.0273427

**Published:** 2022-08-19

**Authors:** Dimitrije Cabarkapa, Michael A. Deane, Andrew C. Fry, Grant T. Jones, Damjana V. Cabarkapa, Nicolas M. Philipp, Daniel Yu

**Affiliations:** Jayhawk Athletic Performance Laboratory–Wu Tsai Human Performance Alliance, University of Kansas, Lawrence, KS, United States of America; University of Castilla-La Mancha, SPAIN

## Abstract

The purpose of the present study was to examine differences in game-related statistical parameters between National Basketball Association (NBA) regular and post-season competitive periods and to determine which variables have the greatest contribution in discriminating between winning and losing game outcomes. The data scraping technique was used to obtain publicly available NBA game-related statistics over a three-year span (2016–2019). The total number of games examined in the present investigation was 3933 (3690 regular season and 243 post-season games). Despite small to moderate effect sizes, the findings suggest that NBA teams’ style of play (i.e., tactical strategies) changes when transitioning from the regular to post-season competitive period. It becomes more conservative (i.e., fewer field goal attempts, assists, steals, turnovers, and points scored), most likely due to greater defensive pressure. Discriminant function analysis correctly classified winning and losing game outcomes during the regular and post-season competitive periods in 82.8% and 87.2% of cases, respectively. Two key game-related statistics capable of discriminating between winning and losing game outcomes were field goal percentage and defensive rebounding, accounting for 13.6% and 14.2% of the total percentage of explained variance during the regular season and 11.5% and 14.7% during post-season competitive periods. Also, overall shooting efficiency (i.e., free-throw, 2-point, and 3-point combined) accounted for 23–26% of the total percentage of explained variance.

## Introduction

Quantitative analysis of game-related statistical parameters has been widely used as a measure of individual and team basketball performance efficiency on various levels of basketball competition [1–15]. Basketball coaches and sport scientists commonly rely on these performance parameters to develop offensive and defensive team strategies, plan off- and on-court training regimens, and identify areas for basketball skill-related improvements [1,2,11,13]. Overall, being able to obtain additional insight into game-related statistical parameters may allow a team to gain a winning edge over the opponent and secure the desired game outcome.

Previous research has found that winning teams in Under-16 junior level men’s basketball competitions exhibited better 2-point and free-throw shooting performance, fewer personal fouls and turnovers, and more assists, steals, and defensive rebounds [1]. Interestingly, no difference between winning and losing teams was found for the number of attempted and made 3-point shots [1]. Over a three-day consecutive competition in the Under-20 competitive level, Ibanez et al. [2] found that winning was mainly attributed to 2-point field goals made, defensive rebounds, and assists. However, winning teams were able to shoot better during the third game from beyond 3-point shooting distances [2]. Although being primarily focused on examining the differences in game-related statistical parameters during close games (i.e., final score difference between 1–9) at the National Collegiate Athletic Association (NCAA) Division-I level of basketball competition, Conte et al. [3] found that winning teams were more likely to attempt less and make more 3-point shots, attempt and make more free-throws, commit fewer personal fouls, and have more defensive rebounds and steals.

When examined on a professional level of European basketball competition (i.e., Hungarian 1^st^ league), winning teams were able to attempt and make more free-throw, 2-point, and 3-point shots as well as have fewer turnovers and more defensive rebounds and assists [4]. Despite each of the previously mentioned game-related statistics playing a crucial role in securing the desired game outcome, defensive rebounds and assists seem to be the top two parameters that differentiate between winning and losing teams during the regular competitive season period in the Spanish ACB league [5,6]. These two performance parameters, along with more blocks and dunks, have also been shown to discriminate in the favor of home-court advantage [7]. It is also important to note that winning teams were capable of finding a scoring opportunity more effectively, both with and without a high level of defensive pressure [8]. Even within top winning teams, defensive rebounds, assists, and personal fouls were capable of successfully discriminating between starting and non-starting players in the Portuguese LPB league [9]. Interestingly, despite these differences, when top winning teams lost, non-starter performance was worse [9]. Moreover, the importance of these game-related statistical parameters seemed to remain persistent even during the post-season competitive period. Winning games during EuroLeague and EuroBasket championship tournaments was mainly attributed to a greater number of defensive rebounds and superior field-goal and free-throw shooting percentage, while the importance of 3-point shooting efficiency became of greater importance during close games [10,11].

While the National Basketball Association (NBA) features some of the best players worldwide and is considered the top level of basketball competition, there is a lack of scientific literature focused on quantitative analysis of game-related statistical parameters related to winning game outcomes during regular and post-season competitive phases, especially over a period of several years. Melnick [12] has found that NBA teams are more likely to be successful when all players commit to passing the ball, causing an increase the team’s total assists. It has also been found that NBA players average more assists and blocks, and fewer steals when compared to European professional basketball players, which may be attributed to their superior athleticism or tactical offensive strategies [13]. During the post-season competitive period, Mateus et al. [14] found that most players tended to take shots more frequently during the first game of the series, while during the last game the number of committed fouls tended to increase, suggesting greater emphasis on defensive actions. In addition, during close games, NBA players tended to focus more on sharing the ball and attempting long-distance shots [14]. In addition, unlike what has been observed on the European level of basketball competition, the game location (i.e., home or away) had no significant impact on variability in NBA players’ performance [15].

Therefore, the purpose of the present study was to (a) examine differences in game-related statistical parameters between the regular and post-season competitive periods, (b) examine differences in game-related statistical performance parameters between winning and losing game outcomes during the regular and post-season competitive periods, and (c) determine which game-related statistical performance parameters have the greatest impact on discriminating between winning and losing game outcomes during the regular and post-season competitive periods on the NBA level of basketball competition.

## Materials and methods

### Data source and procedures

Publicly available NBA game-related statistics were obtained from the Basketball Reference website (*www.basketball-reference.com*) over a three-year span (2016–2019). For each game throughout this competitive period, official box scores (i.e., seasons–summary–schedule–results–box score) were obtained via ParseHub software (North York, ON, Canada). The box scores included the following 18 game-related variables (i.e., team averages) that were included in the data analysis procedures: field goals made (FGM), field goals attempted (FGA), field goal shooting percentage (FG%), 3-point shots made (3PM), 3-point shots attempted (3PA), 3-point shooting percentage (3P%), free-throws made (FTM), free-throws attempted (FTA), free-throw shooting percentage (FT%), offensive rebounds (ORB), defensive rebounds (DRB), total rebounds (TBR), assists (AS), steals (ST), blocks (BL), turnovers (TO), personal fouls (PF), and points (PTS). The total number of games examined in the present investigation was 3933, composed of 3690 regular season and 243 post-season games. Also, due to the public availability of the NBA game-related statistics, the Institutional Review Board’s approval for conducting this project was not needed [16].

### Statistical analysis

Descriptive statistics, means and standard deviations ($\bar{x}$±SD), were calculated for each dependent variable. Independent t-tests were used to examine significant differences in game-related statistical parameters between (a) regular and post-season competitive periods, (b) winning and losing game outcomes during the regular competitive period, and (c) winning and losing game outcomes during the post-season competitive period. Pearson-product moment correlation coefficients (*r*) were used to inspect the relationships between the game-related statistical parameters examined in the present study. A full model multivariate discriminant function analysis was used to examine the magnitude of the relative contribution of each game-related statistical parameter and the ability to classify winning from losing game outcomes, separately for regular and post-season competitive periods. To avoid the issue of multicollinearity, only variables with *r* < 0.60 were entered into the discriminant function analysis model (e.g., FTM is highly correlated with FT% = FGM/FGA x 100%; DRB is highly correlated with TRB = DRB + ORB). Therefore, FTM, FGM, 3PM, TRB, and PTS were eliminated from the discriminant function model. Cohen’s *d* was used to calculate the measure of effect size between game-related parameters examined in the present study (i.e., *d* = 0.2 is a small effect, *d* = 0.5 is a moderate effect, and *d* = 0.8 is a large effect) [17]. Statistical significance was set *a priori* to *p* < 0.05. All statistical analyses were completed with SPSS (Version 26.0; IBM Corp., Armonk, NY, USA).

## Results

Significant differences between the regular and post-season competitive periods were found for all game-related performance parameters except 3PM, 3P%, FT%, DRB, and BL. During the post-season competitive period players had lower FG%, committed more PF, scored fewer PTS, and had fewer FGM, FGA, ORB, TRB, AS, ST, and TO (Table 1).

**Table 1:** Descriptive data ($\bar{x}$±SD) for game-related statistical parameters between the regular and post-season competitive periods.

Game-related statistics	Regular season	Post-season	p-value	Effect size
Field goals made ^†^	39.9 ± 5.2	38.5 ± 5.2	<0.001	0.279
Field goals attempted ^†^	86.9 ± 7.3	84.9 ± 7.5	<0.001	0.257
Field goal percentage ^†^	46.0 ± 5.4	45.4 ± 5.5	0.013	0.115
3-point shots made	10.5 ± 3.7	10.8 ± 3.5	0.089	0.080
3-point shots attempted ^†^	29.4 ± 8.7	30.7 ± 6.9	0.001	0.163
3-point shot percentage	35.8 ± 9.1	35.2 ± 8.6	0.217	0.059
Free-throw shots made ^†^	17.4 ± 6.0	18.4 ± 5.7	<0.001	0.166
Free-throw shots attempted ^†^	22.6 ± 7.3	23.7 ± 6.6	0.002	0.149
Free-throw shot percentage	76.9 ± 10.2	77.4 ± 10.4	0.265	0.052
Offensive rebounds ^†^	10.1 ± 3.7	9.7 ± 3.8	0.038	0.096
Defensive rebounds	34.1 ± 7.1	33.5 ± 5.4	0.110	0.082
Total rebounds ^†^	44.1 ± 6.5	43.2 ± 6.5	0.006	0.128
Assists ^†^	23.5 ± 5.3	22.3 ± 5.2	<0.001	0.220
Steals ^†^	7.7 ± 2.9	7.3 ± 2.8	0.005	0.132
Blocks	4.8 ± 2.5	4.8 ± 2.5	0.752	0.016
Turnovers ^†^	13.5 ± 3.8	12.7 ± 3.8	<0.001	0.224
Personal fouls ^†^	20.2 ± 4.4	21.2 ± 3.8	<0.001	0.244
Points ^†^	107.7 ± 12.5	106.1 ± 12.2	0.005	0.133

^†^: *Statistically significant difference (p < 0*.*05)*.

Besides scoring more PTS, winning teams during the regular season competitive period had greater FG%, 3P%, and FT%, had less FGA, TO, and PF, and had more FGM, 3PM, FTM, FTA, DRB, TRB, AS, ST, BL. No differences were observed for 3PA and ORB between winning and losing teams during the regular season competitive period (Table 2).

**Table 2:** Descriptive data ($\bar{x}$±SD) for game-related statistical parameters between winning and losing teams during the regular season competitive period.

Game-related statistics	Losing teams	Winning teams	p-value	Effect size
Field goals made ^†^	38.0 ± 4.8	41.9 ± 4.8	<0.001	0.822
Field goals attempted ^†^	87.1 ± 7.4	86.7 ± 7.2	0.009	0.062
Field goal percentage ^†^	43.6 ± 4.7	48.4 ± 4.9	<0.001	0.993
3-point shots made ^†^	9.6 ± 3.4	11.4 ± 3.7	<0.001	0.531
3-point shots attempted	29.3 ± 9.8	29.5 ± 7.4	0.276	0.025
3-point shot percentage ^†^	32.7 ± 8.5	38.8 ± 8.6	<0.001	0.719
Free-throw shots made ^†^	16.6 ± 5.8	18.2 ± 6.1	0.001	0.278
Free-throw shots attempted ^†^	21.8 ± 7.0	23.4 ± 7.5	<0.001	0.222
Free-throw shot percentage ^†^	75.9 ± 10.6	77.9 ± 9.7	<0.001	0.203
Offensive rebounds	10.1 ± 3.8	10.0 ± 3.7	0.206	0.027
Defensive rebounds ^†^	32.2 ± 8.3	35.9 ± 5.1	<0.001	0.538
Total rebounds ^†^	42.2 ± 6.3	45.9 ± 6.2	<0.001	0.593
Assists ^†^	21.9 ± 4.7	25.1 ± 5.3	<0.001	0.650
Steals ^†^	7.3 ± 2.8	8.1 ± 2.9	<0.001	0.279
Blocks ^†^	4.4 ± 2.4	5.3 ± 2.6	<0.001	0.334
Turnovers ^†^	14.0 ± 3.9	13.1 ± 3.7	0.001	0.222
Personal fouls ^†^	20.6 ± 4.6	19.8 ± 4.2	<0.001	0.182
Points ^†^	102.0 ± 11.4	113.4 ± 10.9	<0.001	1.020

^†^: *Statistically significant difference (p < 0*.*05)*.

During the post-season competitive period, winning teams scored more PTS, had greater FG%, 3P%, and FT%, had less TO, and more BL, ST, AS, TRB, DRB, FTM, 3PM, and FGM. No significant differences were observed in a number of PF, FTA, 3PA, FGA, and ORB between winning and losing teams during the post-season competitive period (Table 3).

**Table 3:** Descriptive data ($\bar{x}$±SD) for game-related statistical parameters between winning and losing teams during the post-season competitive period.

Game-related statistics	Losing teams	Winning teams	p-value	Effect size
Field goals made ^†^	36.1 ± 4.6	40.8 ± 4.7	<0.001	1.021
Field goals attempted	84.9 ± 7.8	85.0 ± 7.2	0.928	0.008
Field goal percentage ^†^	42.6 ± 4.8	48.1 ± 4.8	<0.001	1.153
3-point shots made ^†^	9.6 ± 3.1	11.9 ± 3.6	<0.001	0.692
3-point shots attempted	30.2 ± 6.7	31.1 ± 7.1	0.128	0.138
3-point shot percentage ^†^	31.9 ± 7.7	38.5 ± 8.2	<0.001	0.823
Free-throw shots made ^†^	17.8 ± 5.7	18.9 ± 5.6	0.040	0.188
Free-throw shots attempted	23.5 ± 6.6	23.9 ± 6.6	0.471	0.065
Free-throw shot percentage ^†^	75.8 ± 10.6	79.0 ± 10.0	<0.001	0.307
Offensive rebounds ^†^	10.1 ± 3.9	9.3 ± 3.6	0.027	0.200
Defensive rebounds ^†^	31.6 ± 4.8	35.4 ± 5.3	<0.001	0.754
Total rebounds ^†^	41.7 ± 6.3	44.8 ± 6.3	<0.001	0.487
Assists ^†^	20.7 ± 4.4	24.0 ± 5.4	<0.001	0.679
Steals ^†^	6.9 ± 2.9	7.7 ± 2.7	0.002	0.283
Blocks ^†^	4.1 ± 2.2	5.5 ± 2.7	0.001	0.549
Turnovers ^†^	13.3 ± 3.5	12.0 ± 3.9	<0.001	0.346
Personal fouls	21.5 ± 3.9	21.0 ± 3.6	0.157	0.128
Points ^†^	99.7 ± 10.1	112.5 ± 10.6	<0.001	1.240

^†^: *Statistically significant difference (p < 0*.*05)*.

See Table 4 for a complete summary of changes in game-related statistical parameters between the regular and post-season competitive phases and winning and losing game outcomes during both competitive phases.

**Table 4:** Summary of differences in game-related performance parameters between (a) regular and post-season competitive periods, (b) winning and losing game outcomes during the regular season competitive period, and (c) winning and losing outcomes during the post-season competitive period. Upward arrow (↑) represents a significant increase, arrow down (↓) represents a significant decrease, and dash (—) represents no statistically significant differences.

Game-related statistics	Regular vs. post-season	Winning vs. losing regular season	Winning vs. losing post-season
Field goals made	↑ 1.4	↑ 3.9	↑ 4.7
Field goals attempted	↑ 2	↓ 0.4	—
Field goal percentage	↑ 0.6%	↑ 4.8%	↑ 5.5%
3-point shots made	—	↑ 1.8	↑ 2.3
3-point shots attempted	↓ 1.3	—	—
3-point shot percentage	—	↑ 6.1%	↑ 6.6%
Free-throw shots made	↓ 1	↑ 1.6	↑ 1.1
Free-throw shots attempted	↓ 1.1	↑ 1.6	—
Free-throw shot percentage	—	↑ 2%	↑ 3.2%
Offensive rebounds	↑ 0.4	—	↓ 0.8
Defensive rebounds	↑ 0.6	↑ 3.7	↑ 3.8
Total rebounds	↑ 0.9	↑ 3.7	↑ 3.1
Assists	↑ 1.2	↑ 3.2	↑ 3.3
Steals	↑ 0.4	↑ 0.8	↑ 0.8
Blocks	—	↑ 0.9	↑ 1.4
Turnovers	↑ 0.8	↓ 0.9	↓ 1.3
Personal fouls	↓ 1	↓ 0.8	—
Points	↑ 1.6	↑ 11.4	↑ 12.8

A full model multivariate discriminant function analysis including 13 game-related statistical parameters with the lowest amount of shared variance (FGA, FG%, 3PA, 3P%, FTA, FT%, ORB, DRB, AS, ST, BL, TO, and PF) was statistically significant for both regular (Λ = 0.576, X^2^[13] = 4069.26, *p* = <0.001) and post-season (Λ = 0.445, X^2^[13] = 386.69, *p* = < 0.001) competitive periods, and were capable of correctly classifying between winning and losing game outcomes in 82.8% and 87.2% of cases, respectively. See Tables 5 and 6 for standardized discriminant function coefficients, percentage of explained variance, and percentage of the total variance.

**Table 5:** Standardized discriminant function coefficients and percentage of explained and total variance for game-related statistical parameters during the regular season competitive period.

Game-related statistics	Standardized coefficients	Percentage of total variance	Percentage of explained variance
Field goal percentage	0.711	16.4	13.6
Defensive rebounds	0.603	13.9	11.5
Offensive rebounds	0.515	11.9	9.8
Turnovers	0.453	10.4	8.6
Steals	0.423	9.7	8.1
Field goals attempted	0.378	8.7	7.2
3-point shot percentage	0.299	6.9	5.7
Blocks	0.208	4.8	4.0
Free-throw shot percentage	0.194	4.5	3.7
Personal fouls	0.191	4.4	3.6
Free-throw shots attempted	0.188	4.3	3.5
3-point shots attempted	0.091	2.1	1.8
Assists	0.086	2.0	1.7
Total		100	82.8

**Table 6:** Standardized discriminant function coefficients, percentage of explained and total variance for game-related statistical parameters during the post-season competitive period.

Game-related statistics	Standardized coefficients	Percentage of total variance	Percentage of explained variance
Defensive rebounds	0.815	16.9	14.7
Field goal percentage	0.787	16.3	14.2
Turnovers	0.598	12.4	10.8
Offensive rebounds	0.494	10.2	8.9
Field goals attempted	0.489	10.1	8.8
Steals	0.433	9.0	7.8
3-point shot percentage	0.317	6.6	5.7
Free-throw shot percentage	0.316	6.6	5.7
3-point shots attempted	0.222	4.5	4.0
Blocks	0.179	3.7	3.3
Personal fouls	0.097	2.0	1.8
Free-throw shots attempted	0.049	1.0	0.9
Assists	0.032	0.7	0.6
Total		100	87.2

## Discussion

### Regular vs. post-season

Based on the differences in game-related statistical parameters, the findings of the present study suggest that the NBA teams’ style of play (i.e., tactical strategies) changes when transitioning from the regular to post-season competitive period. Unlike the regular season where each team plays 82 games, a play-off series consists of four-to-seven games where any unnecessary mistake may jeopardize the team’s chances of securing the desired game outcome and continuing their season. The observed significant decreases in FGM, FGA, and FG% during the post-season may be primarily attributed to an increase in defensive pressure [13,14]. NBA players are likely to become more aggressive closer to the end of the post-season competitive period to preclude the opponent from having uncontested scoring opportunities [14]. Greater defensive pressure may force the opponent to remain in ball possession deeper into the 24-second shot clock, which can eventually lead to fewer FGA, fewer scoring opportunities, and a lower number of total PTS scored during the game. Also, by refraining from sharing the ball, possibly due to a fear of committing an unnecessary mistake, the average number of AS, TO, and ST during the post-season competitive period is notably lower.

### Enhanced defensive pressure

Better defensive cohesiveness may limit near-the-basket dribble-penetration scoring opportunities and force players to attempt more long-range shots [14]. This can explain the significantly greater number of 3PA observed in the present data during the post-season competitive period. Also, the decrease in FG% may be attributed to changes in shooting form under defensive pressure. Gorman & Maloney [18] found that defended shots tended to elicit greater variability in the shooting motion, faster shot execution, longer jump times, and an increase in the amount of time that the ball spent in the air while traveling towards the basket. Overall, these alterations were accompanied by a decrease in shooting accuracy of over 20% [18]. In addition, Sampaio et al. [9] have found that the number of committed PF was one of the main game-related statistical parameters discriminating between starters and non-starters on a European professional level of basketball competition (i.e., Portuguese LPB league). Due to the importance of the game outcome, it is anticipated that the best players (i.e., starters) are more likely to have the ball in their hands and play more minutes during the post-season competitive period. In situations when a better offensive player creates a scoring advantage, the defender might decide to commit PF to stop the opponent from scoring, especially when being guarded by a less skilled/experienced player. Therefore, with an increase in defensive pressure, the best players are more likely to get fouled, which provides a possible explanation for a greater number of PF and FTA observed in the present study during the post-season competitive period.

### Role of shooting efficiency

When examining the difference in game-related statistical parameters between winning and losing teams during both the regular and post-season competitive periods, the winning teams had more FGM, 3PM, and FTM, as well as greater FG%, 3P%, and FT%. These findings suggest that shooting efficiency is of critical importance and that the team that shoots better is most likely going to secure the desired game outcome. Also, these findings are in agreement with Mikolajec et al. [19] who found that performance at the NBA level of basketball competition is primarily determined by offensive scoring parameters. Based on a full model discriminant function analysis, FG%, 3P%, and FT%, accounted for 13.6%, 5.7%, and 3.7% of the total percentage of explained variance during the regular season and 14.2%, 5.7%, and 5.7% during the post-season competitive period, respectively. Combined, 23–26% of the total percentage of the explained variance can be attributed to the team’s shooting efficiency when discriminating between winning and losing teams on the NBA level of basketball competition. It is interesting to note that 3P% accounted for the same percentage of explained variance (i.e., 5.7%), suggesting the equal importance of long-distance shooting efficiency for securing the desired game outcome during both the regular and post-season competitive periods. One of the main advantages of the efficient 3-point shooting performance is that it can allow teams to score the same number of points by attempting fewer field goals and/or having fewer ball possessions. For example, to score 12 points, the team would need to make 6-of-12 2-point attempts compared to 4-of-10 3-point attempts. Similar findings were obtained by Garcia et al. [20] who found that winning teams during the regular season on a European professional level of basketball competition (i.e., Spanish ACB league) dominated in 2-point and 3-point field goals. Although successful shots from long-range shooting distances could have major implications on final score differences, effectiveness in short-distance field goals was of critical importance for securing the winning game outcome during games where the final score difference was less than 12 points [20]. A similar pattern was also observed in the present investigation, where the greatest portion of the explained variance regarding the shooting performance parameters was attributed to the FG%. In addition, Csataljay et al. [11] found that the number of FTM and FT% had a significant contribution to achieving a greater number of scored points and consequently determined successful performance during the post-season competitive period. While offering further support to the findings of the present study, it is important to note that no difference in FTA was observed between winning and losing NBA teams during the post-season competitive period. Winning teams were capable of attaining greater FT% and having more FTM for the same number of FTA, further emphasizing the importance of shooting efficiency for securing the desired game outcome.

### Role of defensive rebounds and blocks

Alongside superior shooting efficiency, winning teams had more DRB and BL during both the regular and post-season competitive periods. Being able to secure more DRB reduces the overall number of scoring opportunities and second-point chances for the opposing team. The findings of the present study are in the agreement with previously conducted scientific literature emphasizing the importance of DRB as one of the key parameters capable of distinguishing between winning and losing game outcomes on various levels of basketball competition [2,5,6,10,21]. Based on the discriminant function analysis, DRB accounted for 11.5% and 14.7% of the total explained variance during the regular and post-season competitive periods, respectively. An increase in the importance of DRB during the post-season competitive period may be primarily attributed to a greater emphasis on defensive performance intensity [14], as every unnecessary mistake might jeopardize the team’s chances of securing the winning game outcome and continuing their season. The teams that frequently won post-season games on a professional level of European basketball competition showed better tactical discipline and responsibility in controlling defensive positions, such as boxing-out the opponent to secure a defensive rebound and minimizing the number of handicap positions [10]. Alongside better DRB performance, the findings of the present study indicated that winning teams had more BL, as another defensive tool/skill capable of preventing the opponent from having uncontested scoring opportunities. Although not reaching the level of statistical significance, similar results emerged when examining teams on a top European professional level of basketball competition (i.e., Spanish ACB league) where winning teams committed more and received fewer blocks [20]. Furthermore, it has been found that NBA players outperform their Euroleague peers in blocking performance, which may be mainly attributed to differences in anthropometric characteristics (i.e., NBA players are taller and heavier) and the overall style of play [22].

### Role of ball handling

Based on the findings of the present study, it is noted that NBA winning teams have a significantly greater number of AS and ST, and fewer TO during both the regular and post-season competitive periods. A greater number of AS suggests that winning teams are sharing the ball more efficiently which may allow them to secure more uncontested scoring opportunities and ultimately attain greater FG% [12]. Ibanez et al. [23] found that professional players (i.e., expert players) have greater offensive control and are able to execute more collective actions and find better shooting positions. This may be of critical importance in close games, especially during the post-season period, where the ball is supposed to be assisted at the right time to the best player on the court capable of shooting with high confidence and accuracy. On the other hand, inaccurate passes can lead to TO and ST which can diminish the team’s chances of securing the desired game outcome. If a player can steal the ball, it allows the opposing team to regain offensive control over the ball and allows for an additional scoring opportunity, most likely an open scoring opportunity if the ST was committed further from the opposing team’s basket. These findings are in the agreement with the previously conducted scientific literature [3,4,24]. It has been found that winning teams on the NCAA Division-I level of basketball competition are likely to have a higher number of ST when compared to the losing teams [3]. Likewise, when examining the performance difference between winning and losing teams on a European professional level of basketball competition, winning teams had fewer TO and more ST across all games as well as during unbalanced games characterized by a difference in the final score between 12–22 points [4]. Moreover, Sampaio et al. [24] reported that besides shooting performance, recovered balls (i.e., TO, ST, and BL) were the biggest contributors to point differences in basketball games played during the 2008 Beijing Olympic Games. Thus, the ability to recover more balls from the opponent and convert them into effective scoring opportunities was one of the main characteristics attributed to the USA’s dominance [24].

### Role of personal fouls and tactical strategies

Interestingly, no difference between winning and losing teams was observed in the number of committed PF during the post-season competitive period, while winning teams had a significantly lower number of ORB. As previously indicated, the importance of tactical play and defensive performance increases during the post-season competitive period when winning becomes more important [13,14]. The best players (i.e., starters) are more likely to play greater minutes and have the ball in their hands more often because they are capable of making fast offensive decisions in assisting the ball to a player in a favorable position to score [9]. Therefore, due to an increase in defensive pressure, the likelihood of the best players getting fouled on both teams might be the same, which may provide a possible explanation for no difference in the number of PF observed between winning and losing teams during the post-season competitive period. In addition, the difference in ORB may be attributed to changes in the tactical strategies of the losing teams. In NBA, centers are commonly specialized to complete offensive rebounding tasks [21], while the rest of the players tend to quickly return on defense to prevent the opponent’s fast-break scoring opportunities. This strategy might be susceptible to changes in situations when the losing team is trailing and trying to come back in the game. Csataljay et al. [25] have found that the efficiency of the offensive rebounding was primarily influenced by the number of players that decided to perform the offensive rebounding task. Thus, to make up for the difference in scoring margin, the losing team might send more players (e.g., center and power-forward or center and small-forward) to crash the offensive board in order to pursue more second-point scoring opportunities. On the other hand, an increase in ORB during the post-season competitive phase could also be attributed to the winning team purposely allowing ORB close to the end of the game by returning 4–5 players on defense. This is most likely to occur when the difference in the scoring margin is large and where it might be hard for the opposing team to catch up.

### Practical applications

The findings of the present study may help basketball coaches to develop game tactics (e.g., offensive and defensive strategies) that adequately resemble the demands of the regular and post-season competitive periods and optimize the training process by selecting basketball-specific drills targeted toward improvements in shooting accuracy and rebounding. For example, focus on practicing shooting motions with the presence of a defender, and focus on practicing boxing-out and anticipating ball trajectory during rebounding drills. Also, the findings of the present study may benefit basketball scouts when selecting players with a skill set that aligns with on-court playing demands at the NBA level of competition. For example, selecting a player that is an excellent shooter with great rebounding skills.

## Limitations

Although the present study provides valuable information regarding some of the key game-related statistical parameters associated with winning game outcomes during the regular and post-season competitive periods, there are some limitations that should be acknowledged. The data scraping technique has its limitations related to the reliability of the data obtained from publicly available sources. Also, the location of the game (e.g., home or away), playing position, injury status, and the number of games and minutes played by each player were not included in the present analysis, which are important factors that need to be considered and warrant further investigation.

## Conclusion

In conclusion, based on the observed differences in game-related statistical parameters, the findings of the present study suggest that NBA teams’ style of play (i.e., tactical strategy) changes when transitioning from the regular to post-season competitive period. It becomes more conservative (i.e., fewer field goal attempts, assists, steals, turnovers, and points scored), most likely due to greater defensive pressure. FG% and DRB are two key game-related statistical parameters capable of discriminating between winning and losing game outcomes during both the regular and post-season competitive periods. FG% and DRB accounted for 13.6% and 14.2% of the total percentage of explained variance during the regular season and 11.5% and 14.7% during post-season competitive periods, respectively. Overall shooting efficiency (i.e., free-throw, 2-point, and 3-point combined) accounted for 23–26% of the total explained variance. Thus, by attaining peak shooting efficacy and defensive rebounding performance NBA teams may increase the likelihood of securing the desired game success.
